# Estimating the economic burden of respiratory syncytial virus infection among children <2 years old seeking care in North-West Nigeria

**DOI:** 10.7189/jogh.15.04307

**Published:** 2025-12-12

**Authors:** Farina Leonie Shaaban, Maria Ahuoiza Garba, An Nguyen, Clint Pecenka, Neele Rave, Louis J Bont

**Affiliations:** 1 Department of Paediatrics, University Medical Centre Utrecht, Utrecht, the Netherlands; 2 Department of Paediatrics, Ahmadu Bello University Teaching Hospital, Zaria, Nigeria; 3 Center for Vaccine Innovation and Access, PATH, Ho Chi Minh City, Vietnam; 4 Center for Vaccine Innovation and Access, PATH, Seattle, Washington, USA

## Abstract

**Background:**

Respiratory syncytial virus (RSV) disease burden is highest in low- and middle-income countries; yet data for costs of RSV-related illness in these settings are scarce. We estimated RSV costs of illness to inform decision-making for RSV preventive strategies in Nigeria.

**Methods:**

This prospective study assessed the costs of care per episode of (severe) acute respiratory infection during one RSV season (April to November 2023). Children <2 years old, were recruited at tertiary inpatient and outpatient facilities in North-West Nigeria and grouped as non-severe (outpatient), severe (inpatient non-intensive care), and life-threatened (intensive care or fatality) cases. Direct medical, direct non-medical, and indirect cost data were collected using caregiver questionnaires at the index visit or day of admission and follow-up and gathered from hospital records at discharge. We summarised average costs in 2023 USD.

**Results:**

We included 792 children (mean age 8.7 months) with non-severe (n = 692), severe (n = 52), and life-threatening (n = 48) respiratory infections. Among these groups we confirmed RSV infection in 130 (19%), 19 (37%), and 14 (29%) children. The average societal costs were USD 13 (95% confidence interval (CI) = 11–14), USD 244 (95% CI = 198–290), and USD 179 (95% CI = 120–239) per episode of non-severe, severe, and life-threatening RSV infection, respectively. Costs associated with RSV did not increase stepwise with the severity categories due to small group sizes and fatalities with low costs included in the life-threatened group. For severe RSV, average household costs were over 200% of the national monthly minimum wage. Most households (89%) resorted to personal savings to cover costs.

**Conclusions:**

In young children in Nigeria, RSV presented a significant health and economic burden. This study can inform health economic evaluations of emerging RSV pharmaceutical interventions for Nigeria and may be adjusted for rural/urban contexts and level of care.

Respiratory syncytial virus (RSV) is the primary pathogen associated with acute lower respiratory tract infections (LRTIs) in early childhood. Globally, it is estimated that RSV is associated with 33 million infections and over 100 000 deaths annually in children <5 years old. This burden disproportionately affects low- and middle-income countries (LMICs), where 97% of deaths attributed to RSV occur [1].

Infant and under-five mortality rates remain high in Nigeria at 67 and 132 deaths per 1000 live births, respectively, reported in 2018 [2]. In Nigeria, the country with the highest number of under-five pneumonia deaths reported worldwide, LRTIs are a leading cause of childhood morbidity and mortality [3], and RSV contributes substantially to this burden. One in three children aged 1 through 59 months admitted to hospital with severe respiratory symptoms tested positive for RSV [4], as well as 30% of children <2 years old receiving intensive care for respiratory symptoms at the same tertiary hospital in North-West Nigeria [5]. Community surveillance studies have also highlighted the burden of childhood RSV mortality in Nigeria (74 per 100 000 child-years in children <5 years old [6].

Novel pharmaceutical interventions to prevent RSV-associated illness are entering the market, with many more in development [7]. Two such strategies have been approved and introduced in some countries. Nirsevimab is a long-acting monoclonal antibody (mAb) administered to infants at birth or the start of the season, and the bivalent RSVpreF vaccine is a maternal vaccine given during the third trimester of pregnancy [8,9]. Both strategies showed promising trial results to protect infants against medically attended RSV infections during their first RSV season [10,11], and have been recommended for global implementation [12].

To inform decision-making on the potential prioritisation and implementation of these interventions, country-specific data of the burden of RSV and associated costs of care are critical. Globally, RSV is estimated to cost close to USD 5 billion annually, and detailed data are available for high-income countries [13]. Such data, however, are lacking for most LMICs, including Nigeria. This study describes the direct medical and non-medical costs along with indirect costs associated with RSV infections among children <2 years old seeking care in North-West Nigeria from the perspectives of the households, health system, and the society overall.

## METHODS

### Study setting

This study was part of the RSV Global Online Mortality Database (GOLD) III – Health Economics Study conducted in four countries eligible for support from Gavi, The Vaccine Alliance [14,15], as an extension of the RSV GOLD III–ICU Network Study [5]. The current study was conducted at the Ahmadu Bello University Teaching Hospital (ABUTH) and its associated public outpatient clinic, Institute of Child Health (ICH) Banzazzau, in North-West Nigeria. The study facility, ABUTH, is one of the largest public teaching hospitals in Nigeria and is situated rurally in the outskirts of the ancient city of Zazzau and serves as a referral centre for the North-Western states of Nigeria, home to a quarter of the country’s population.

The Nigerian health system is characterised by both public and private facilities providing primary, secondary, and tertiary care services. The government plays a dual role, overseeing public and private facilities. Nigerian households bear substantial health care costs. In 2021, 76% of health care expenditures were out-of-pocket [16]. A small fraction (5%) of Nigerians (most of whom are employed in the formal sector) benefits from the national health insurance scheme (NHIS) [17].

### Study population

Children <2 years old presenting with (severe) acute respiratory illness ((S)ARI) at ABUTH and ICH were eligible for inclusion. Neonates less than four days old were excluded from the study. Participating children were categorised into the following severity groups: non-severe (outpatient care), severe (inpatient care, non-intensive care unit (ICU)), and life-threatened (ICU admission or fatality). This study was granted ethical approval by the Health Research Ethics Committee (#954524802) of the Ahmadu Bello University Teaching Hospital Shika, Zaria, Nigeria, and the Medical Research Ethics Committee NedMec (#20–536) of the University Medical Centre in Utrecht, The Netherlands.

### Study timeline

This study spanned one local respiratory season from April 2023 to November 2023. Recruitment efforts experienced two disruptions during this time. First, recruitment was minimal from the 26th of July to the 20th of August, due to a resident doctors’ strike. Second, testing ended two weeks early due to depleted testing materials. We had not detected RSV in the two weeks prior to testing cessation; therefore, the season was assumed to be over.

### Data collection

Once caregivers gave consent to participate in the study, a nasopharyngeal sample was taken from eligible children (at index visit or day of admission) and tested within 72 hours. We determined RSV infection status using a molecular point-of-care test ID Now RSV (Abbott, Scarborough, ME, USA) [18]. Attending physicians and caregivers were informed of the test result. To allow for comparisons, children were included in the study regardless of RSV status.

Trained staff collected data during questionnaire-led caregiver interviews conducted at multiple time points. Caregiver questionnaires administered at the index visit or on the day of admission measured participating children’s socioeconomic and clinical characteristics and ascertained costs associated with prior care and the index visit or admission. Detailed billing data were extracted from patient records once patients were discharged. Additional out-of-pocket costs were captured during the follow-up interviews. Follow-up interviews were conducted to ascertain subsequent care and costs related to the same episode of illness and took place by telephone four to six weeks post-visit or discharge (Figure S1 in the **Online Supplementary Document**). When caregivers were unable to present receipts or recall costs, facility pricing was assumed. Data collectors captured all information on hardcopy and later entered them in a Castor electronic data capture system [19].

### Cost data

We collected cost data that fell within three categories. Direct medical costs (*e.g*. fees, medications, laboratory services), direct non-medical costs (*e.g*. transport, meals, lodging), and indirect costs (lost income and lost leisure measured as a product of time lost and reported income or minimum wage, respectively). We described costs of illness from the perspectives of the health system and households, the sum of which was presented as the societal perspective. From the household perspective, costs included out-of-pocket direct medical costs, direct non-medical costs, and indirect costs. Cost data were collected in Nigerian Naira (NGN) and converted to US dollars (average 2023 exchange rate: USD 1 = NGN 591.37) [20].

#### Health system perspective

Health system costs were the difference between the total cost and out-of-pocket costs. They included direct medical costs covered by NHIS and overhead expenses. For each itemised direct medical cost, we recorded the amount paid out-of-pocket and by NHIS. Overhead expenses consisted of facility expenses and wage costs. Annually, ABUTH is allocated fixed federal funding for facility expenses including wages for supporting staff (*e.g*. janitorial and security staff). Due to limited access to departmental financial records, we used hospital budgetary guidelines to estimate the portion of federal funding allocated to the paediatric inpatient wards at ABUTH and the ICH during the study period. We estimated clinical staff wages separately based on federal wage brackets per employment cadre, and average daily staffing per participating ward and outpatient clinic. For outpatients, overhead expenses were estimated per patient based on the total number of children that visited the ICH during the study period. For inpatients, we estimated the total number of patient days spent at the participating wards, assuming a seven-day median length of stay (based on the current study). Inpatient overhead expenses were expressed as costs per patient day and multiplied by the individual length of stay to estimate the total cost per admission for each child.

### Data analysis

We described the demographic, socioeconomic, and clinical characteristics of included children and summarised the costs associated with an episode of respiratory illness. We ascertained total costs of care utilisation using a bottom-up costing approach, whereby the unit cost of each service and product was multiplied by the frequency of use and summed. Costs were summarised as mean (with zero censored 95% confidence interval (CI) bootstrapped over 1000 replications) and median (with interquartile range (IQR)). Normality was assessed visually, and we used Wilcoxon rank-sum and Kruskal-Wallis tests to compare the distributions of characteristics and costs across patient groups. Total costs were the sum of all direct medical, direct non-medical, and indirect costs. Analyses were conducted using Stata version 17.0 (Stata Corporation LLC, College Station, TX, USA) and *R*, Studio version 24.04 (R Foundation for Statistical Computing, Vienna, Austria).

#### Sensitivity analysis

We conducted stepwise analyses whereby cases were excluded for three reasons to explore costs without the effects of costs observed at the extremes (high or low by definition). First, we excluded children admitted for 10 days or longer (n = 24 excluded). This sensitivity analysis was independent of the following two. Second, we excluded all children that were missing billing information (n = 15 excluded). Finally, we extended the second step and additionally excluded outpatient fatalities (n = 17 excluded).

Due to its rural setting, ABUTH frequently experiences a low bed occupancy rate. We conducted two independent sensitivity analyses whereby we adjusted the overhead expense calculations to explore the representativeness of our study findings for Nigerian tertiary health care facilities. First, we assumed 100% bed occupancy instead of observed patient numbers to estimate per inpatient bed day overhead expenses. Second, we used the World Health Organization CHOosing Interventions that are Cost-Effective (WHO CHOICE) estimates of costs for health service delivery at tertiary facilities as a proxy for overhead expenses [21]. The WHO CHOICE cost per bed day in NGN was adjusted for inflation (2010 to 2023) and converted to 2023 USD.

## RESULTS

### Patient characteristics

Overall, 804 children were recruited. Due to lost records, 792 were included in analyses, of which 88% (n = 696) sought outpatient care and the rest were admitted to the paediatric ward (7%, n = 54) or intensive care unit (5%, n = 42). We detected RSV in 21% (n = 163) of all participating children, and we described one full local RSV season (Figure S2 in the **Online Supplementary Document**). Of the 163 RSV-positive cases, 80% (n = 130) were non-severe, 12% (n = 19) were severe, and 9% (n = 14) were life-threatened. Of the 629 RSV-negative cases, 89% (n = 562) were non-severe, 5% (n = 33) were severe, and 5% (n = 34) were life-threatened (**Table 1**).

**Table 1:** Demographics and clinical characteristics of RSV-positive and RSV-negative children <2 years old, by severity level*

	RSV positive				RSV-negative					All patients	
	**Total**	**Non-severe**	**Severe**	**Life-threatened**		**Total**	**Non-severe**	**Severe**	**Life-threatened**		**Total**
Total number of participants	163	130	19	14		629	562	33	34		792
Age in months, x̅ (SD)	6.5 (5.7)	7.0 (5.8)	4.5 (4.8)	5.1 (4.5)		9.3 (5.9)	9.3 (5.9)	8.0 (7.4)	10.3 (5.6)		8.7 (6.0)
Female	73 (44.8)	58 (44.6)	4 (21.1)	11 (78.6)		291 (46.3)	255 (45.4)	17 (51.5)	19 (55.9)		364 (46.0)
Household size, MD (IQR)	6 (4–9)	5 (4–8)	8 (6–15)	6 (4–7)		5 (4–8)	5 (4–8)	7 (5–13)	7 (3–10)		5 (4–8)
Level of education primary caregiver secondary school or higher	140 (85.9)	109 (83.9)	17 (89.5)	14 (100)		540 (85.9)	493 (87.7)	21 (63.6)	26 (76.5)		680 (85.9)
Distance to healthcare facility in km, MD (IQR)	8 (5–13)	8 (5–10)	19 (15–22)	11 (7–22)		8 (4–12)	7 (3–10)	15 (8–22)	15 (10–20)		8 (4–12)
Diagnosis at admission											
*Bronchiolitis*	4 (2.5)	-	2 (10.5)	2 (15.4)		3 (0.5)	-	1 (3.1)	2 (6.3)		7 (0.9)
*Pneumonia*	24 (14.8)	-	17 (89.5)	7 (53.9)		38 (6.1)	10 (1.8)	13 (40.6)	15 (46.9)		62 (7.9)
*URTI*	104 (64.2)	101 (77.7)	-	3 (23.1)		420 (67.6)	413 (74.2)	3 (9.4)	4 (12.5)		524 (66.9)
*Other*	30 (18.5)	29 (22.3)	-	1 (7.7)		160 (25.8)	134 (24.1)	15 (46.9)	11 (34.4)		190 (24.3)
Prematurity	4 (2.5)	3 (2.3)	-	1 (7.1)		12 (1.9)	9 (1.6)	-	3 (8.8)		16 (2.0)
Comorbidity	71 (43.6)	68 (52.3)	1 (5.3)	2 (14.3)		286 (45.5)	267 (47.5)	9 (27.3)	10 (29.4)		357 (45.1)
Length of stay in days, MD (IQR)	7 (5–9)	N/A	7 (5–8)	7 (4–11)		6 (5–10)	N/A	6 (5–9)	7 (5–11)		7 (5–10)
Previous medical consultation	30 (18.4)	19 (14.6)	6 (31.6)	5 (35.7)		160 (25.4)	135 (24.0)	11 (33.3)	14 (41.2)		190 (24.0)
Follow-up care	15 (9.4)	9 (6.9)	4 (23.5)	2 (16.7)		61 (9.8)	55 (9.9)	2 (6.1)	4 (13.3)		76 (9.8)
Mortality	3 (1.9)	-	-	3 (21.4)		7 (1.1)	-	-	7 (20.6)		10 (1.3)

IQR – interquartile range, MD – median, RSV – respiratory syncytial virus, SD – standard deviation, URTI – upper respiratory tract infection, x̄ – mean

*Values presented as n (%) unless specified otherwise.

Overall, fewer female children were included in the study than male (46%, n = 364); however, the life-threatened RSV-positive and RSV-negative cases were mostly female children (79%, n/N = 11/14 and 56%, n/N = 19/34, respectively). The average age was 8.7 months (standard deviation (SD) = 6 months), and RSV-positive children were younger than RSV-negative children (6.5 *vs*. 9.3 months, *P* < 0.001). Children admitted to ABUTH were more likely to live farther away than children visiting ICH (*P* < 0.001), and travelled distance significantly differed across severity groups (*P* < 0.001). The median length of stay was seven days (IQR = 5–10 days). Most caregivers were the child’s mother (98%, n = 774) and had at least secondary school level education (86%, n = 680). We recorded ten fatalities (RSV-positive, n = 3), including in-hospital (n = 3) and community deaths (n = 7). Community deaths occurred after receiving outpatient care only (outpatient fatalities, n = 4) and after receiving inpatient care (post-discharge fatalities, n = 3).

### Cost per episode

#### Societal costs

On average, one episode of RSV-related illness cost USD 13 (95% CI = 11–14) for non-severe cases, USD 244 (95% CI = 198–290) for severe cases, and USD 179 (95% CI = 120–239) for life-threatened cases (**Table 2**). The inclusion of fatalities in the life-threatened group impacted the estimated average costs, regardless of level of care services received. This effect was stronger in RSV-positive than RSV-negative cases due to small group sizes for RSV-positive cases and resulted in lower costs estimated per episode of life-threatening RSV than severe RSV. This observation was consistent across the analyses outlined below. The costs of outpatient *vs*. inpatient RSV cases were USD 13 (95% CI = 11–14) and USD 230 (95% CI = 193–267), respectively (Table S1 in the **Online Supplementary Document**). Average costs for the index visit or admission alone (excluding prior and follow-up care) were USD 11 (95% CI = 11–11), USD 231 (95% CI = 188–275), and USD 176 (95% CI = 117–235), for non-severe, severe, and life-threatening RSV, respectively (data not shown).

**Table 2:** Summary of costs per LRTI episode among children <2 years old, by RSV status and severity, expressed in 2023 USD

	RSV-positive (n = 163)			RSV-negative (n = 629)		
	**Non-severe (n = 130)**	**Severe (n = 19)**	**Life-threatened (n = 14)**	**Non-severe (n = 562)**	**Severe (n = 33)**	**Life-threatened (n = 34)**
**Societal costs total**						
x̅ (95% CI)	12.68 (11.20–14.16)	244.07 (197.77–290.36)	179.36 (120.23–238.50)	18.23 (16.31–20.15)	237.75 (205.32–270.18)	279.52 (201.23–357.81)
MD (IQR)	11.30 (9.37–13.39)	209.31 (188.34–289.22)	184.36 (117.17–278.21)	12.08 (9.96–16.44)	208.83 (196.56–271.93)	208.50 (166.69–327.82)
Direct medical costs						
x̅ (95% CI)	10.49 (9.11–11.87)	222.44 (179.24–265.65)	152.57 (101.34–203.81)	13.68 (12.37–15.00)	210.71 (185.20–236.23)	224.56 (173.38–275.74)
MD (IQR)	9.08 (7.85–11.23)	196.02 (176.11–252.20)	155.94 (95.89–239.66)	9.65 (8.27–13.13)	190.52 (167.35–257.68)	178.70 (142.77–262.17)
*Hospitality/ facility-based fees*						
x̅ (95% CI)	3.99 (3.74–4.23)	136.33 (107.96–164.71)	116.94 (78.66–155.21)	4.18 (4.01–4.36)	137.90 (118.67–157.13)	152.91 (115.24–190.58)
MD (IQR)	4.05 (3.62–4.05)	130.44 (98.07–141.83)	131.28 (65.37–164.24)	4.05 (3.62–4.05)	115.52 (98.92–148.31)	121.25 (98.07–181.27)
*Medication costs*						
x̅ (95% CI)	6.14 (5.37–6.91)	43.69 (30.21–57.18)	23.27 (15.99–30.55)	8.33 (7.50–9.16)	32.19 (24.33–40.05)	38.64 (25.80–51.47)
MD (IQR)	5.07 (4.14–7.10)	30.27 (19.78–55.97)	22.83 (10.48–29.59)	5.92 (4.31–8.79)	27.44 (15.73–43.33)	24.48 (14.80–41.94)
*Laboratory costs*						
x̅ (95% CI)	1.01 (0.00–2.39)	13.08 (10.74–15.42)	12.84 (8.87–16.80)	1.58 (0.90–2.25)	14.15 (11.03–17.28)	13.89 (10.93–16.85)
MD (IQR)	0.00 (0.00–0.00)	13.53 (9.30–17.76)	10.99 (9.30–16.91)	0.00 (0.00–0.00)	12.26 (9.30–16.49)	11.41 (6.64–19.45)
*Imaging costs*						
x̅ (95% CI)	-	6.81 (5.13–8.49)	6.23 (3.98–8.47)	1.31 (0.21–2.42)	7.58 (5.88–9.28)	6.58 (5.02–8.14)
MD (IQR)	-	6.76 (4.23–9.30)	5.92 (4.23–6.76)	0.00 (0.00–0.00)	6.76 (4.23–10.15)	6.55 (3.38–10.15)
*Procedure costs*						
x̅ (95% CI)	0.74 (0.00–2.17)	12.03 (7.92–16.15)	6.76 (4.26–9.25)	1.04 (0.16–1.91)	13.35 (9.15–17.54)	8.57 (4.97–12.17)
MD (IQR)	0.00 (0.00–0.00)	11.58 (6.51–13.27)	8.20 (0.59–10.74)	0.00 (0.00–0.00)	10.44 (5.92–15.94)	5.66 (0.59–13.27)
*Miscellaneous costs*						
x̅ (95% CI)	-	13.21 (7.56–18.86)	5.94 (1.20–10.68)	0.36 (0.05–0.67)	10.50 (6.35–14.65)	8.59 (6.22–10.96)
MD (IQR)	-	10.78 (5.07–16.66)	3.76 (0.00–9.98)	0.00 (0.00–0.00)	6.21 (5.07–8.79)	7.91 (4.57–11.29)
Direct non-medical costs*						
x̅ (95% CI)	1.69 (1.44–1.95)	13.81 (5.59–22.03)	16.54 (9.71–23.38)	3.13 (2.38–3.89)	16.12 (10.42–21.82)	42.05 (12.51–71.59)
MD (IQR)	1.35 (1.01–1.82)	5.07 (1.95–23.67)	12.13 (5.24–30.28)	1.35 (0.73–2.22)	6.84 (2.38–27.22)	14.44 (3.38–36.99)
*Transport costs*						
x̅ (95% CI)	1.59 (1.39–1.80)	3.42 (2.53–4.32)	2.78 (1.96–3.60)	2.67 (2.07–3.27)	3.40 (2.28–4.52)	8.49 (2.42–14.56)
MD (IQR)	1.35 (1.01–1.77)	3.17 (1.56–5.07)	2.03 (1.35–4.06)	1.35 (0.73–2.03)	2.38 (1.69–4.40)	3.10 (1.74–6.76)
*Meal costs*						
x̅ (95% CI)	0.02 (0.00–0.06)	11.61 (2.90–20.32)	14.67 (7.71–21.63)	0.26 (0.00–0.56)	11.23 (5.84–16.62)	34.32 (5.91–62.73)
MD (IQR)	0.00 (0.00–0.00)	0.00 (0.00–20.29)	11.84 (3.38–25.36)	0.00 (0.00–0.00)	1.69 (0.00–17.76)	12.68 (0.00–20.29)
Indirect costs						
x̅ (95% CI)	1.01 (0.80–1.22)	9.89 (5.85–13.94)	15.94 (8.46–23.42)	1.94 (1.46–2.42)	17.16 (8.21–26.11)	16.26 (8.18–24.35)
MD (IQR)	0.88 (0.59–1.18)	11.80 (0.00–17.40)	16.52 (5.90–28.13)	0.88 (0.59–1.47)	12.68 (9.44–16.52)	9.44 (2.70–20.25)
*Lost income*						
x̅ (95% CI)	0.04 (0.00–0.10)	1.42 (0.00–3.40)	0.98 (0.00–2.14)	0.68 (0.36–0.99)	2.01 (0.38–3.63)	2.74 (0.29–5.18)
MD (IQR)	0.00 (0.00–0.00)	0.00 (0.00–0.00)	0.00 (0.00–0.06)	0.00 (0.00–0.00)	0.00 (0.00–0.00)	0.00 (0.00–1.24)
*Lost leisure*						
x̅ (95% CI)	0.46 (0.34–0.57)	6.40 (2.82–9.97)	9.27 (3.58–14.95)	0.73 (0.61–0.85)	8.92 (3.39–14.45)	10.18 (4.93–15.43)
MD (IQR)	0.00 (0.00–0.88)	0.44 (0.00–15.34)	2.36 (0.00–19.76)	0.59 (0.00–0.88)	2.36 (0.00–12.68)	4.13 (0.00–11.97)
**Health system costs total†**						
x̅ (95% CI)	3.65 (3.59–3.71)	121.86 (89.53–154.19)	91.75 (56.77–126.72)	3.63 (3.62–3.65)	118.43 (100.13–136.74)	133.37 (98.24–168.50)
MD (IQR)	3.62 (3.62–3.62)	110.32 (78.80–126.08)	94.56 (47.28–142.51)	3.62 (3.62–3.62)	94.56 (78.80–141.83)	102.44 (78.80–173.35)
Specified: Hospitality/facility-based fees						
x̅ (95% CI)	3.62 (3.62–3.62)	119.62 (89.84–149.39)	98.75 (63.52–133.98)	3.62 (3.62–3.63)	118.43 (100.13–136.74)	132.78 (97.66–167.90)
MD (IQR)	3.62 (3.62–3.62)	110.32 (78.80–126.08)	110.32 (47.28–141.83)	3.62 (3.62–3.62)	94.56 (78.80–141.83)	102.44 (78.80–173.35)
**Household costs total‡**						
x̅ (95% CI)	9.03 (7.54–10.51)	122.21 (94.38–150.03)	87.62 (61.20–114.04)	14.59 (12.67–16.51)	119.32 (98.45–140.19)	146.15 (97.75–194.55)
MD (IQR)	7.67 (5.75–9.76)	114.17 (79.73–146.91)	82.34 (69.89–135.70)	8.45 (6.34–12.81)	105.19 (87.92–130.03)	103.08 (72.34–154.47)
Specified: Direct medical costs						
x̅ (95% CI)	6.84 (5.45–8.22)	100.59 (79.39–121.79)	60.83 (42.66–79.00)	10.10 (8.78–11.43)	95.16 (79.71–110.62)	91.19 (71.66–110.71)
MD (IQR)	5.45 (4.23–7.61)	100.70 (65.86–126.91)	60.92 (48.62–80.58)	6.09 (4.65–9.55)	82.52 (69.58–108.81)	81.78 (54.87–110.00)

CI – confidence interval, IQR – interquartile range, LRTI – lower respiratory tract infection, MD – median, RSV – respiratory syncytial virus, x̄ – mean

*Besides the listed costs, some cases also reported caregiver (n = 18 / 792) and lodging (n = 6 / 792) costs. Due to few reports, these costs are not shown in the table.

†Health system costs consisted of direct medical costs only and were made up of hospitality/facility-based fees (overhead expenses) and charges covered by the national health insurance scheme (NHIS). Due to very low NHIS coverage (0.8%), these costs were not included separately in the table.

‡Household costs also included direct non-medical and indirect costs, which are reflected in the respective rows under societal costs.

Direct medical costs were the largest contributor to total costs. For severe and life-threatening illness, hospitality/facility-based fees formed the largest cost sub-category; while, for non-severe illness, most costs were for medications and remedies. Overall, total societal costs were higher for the RSV-positive group (USD 54; 95% CI = 39–69) than the RSV-negative group (USD 44; 95% CI = 36–52), but the difference was not statistically significant (*P* = 0.35, data not shown).

#### Health system costs

NHIS coverage was only reported for six children (0.8%). Therefore, health system costs consisted mostly of hospitality/facility-based fees. For non-severe RSV, health system costs were USD 4 (95% CI = 4–4) per visit. Health system costs for severe and life-threatening RSV varied with length of stay and averaged USD 122 (95% CI = 90–154) and USD 92 (95% CI = 57–127), respectively.

#### Household costs

While the health system bore the most cost for hospitality/facility-based fees, households bore the remaining direct medical costs for medication, laboratory and imaging services, and procedures. Total average household costs were USD 9 (95% CI = 8–11), USD 122 (95% CI = 94–150), and USD 88 (95% CI = 61–114) per episode of non-severe, severe, and life-threatening RSV, respectively. Direct medical costs contributed the most to household costs as compared to direct non-medical and indirect costs. The most reported method of payment was personal savings (89% of households; n = 703 data not shown).

#### Sensitivity analysis

When children admitted for 10 days or longer were excluded, total average costs decreased for both those with severe RSV (from USD 244 to USD 217) and life-threatening RSV (from USD 179 to USD 135; Table S2 in the **Online Supplementary Document**). The average societal costs estimated in the main analysis were lower for the group with life-threatening RSV than the group with severe RSV; however, the difference was not statistically significant (*P* = 0.13). When we descriptively explored this difference by excluding children with missing billing data and outpatient fatalities, we observed a rise in average costs in the life-threatened group; whereby the overlap of the 95% CI estimated for children with severe (USD 198–290) and life-threatening (USD 177–274) RSV increased. Due to small group sizes, however, these results should be interpreted with care.

**Figure 1:**
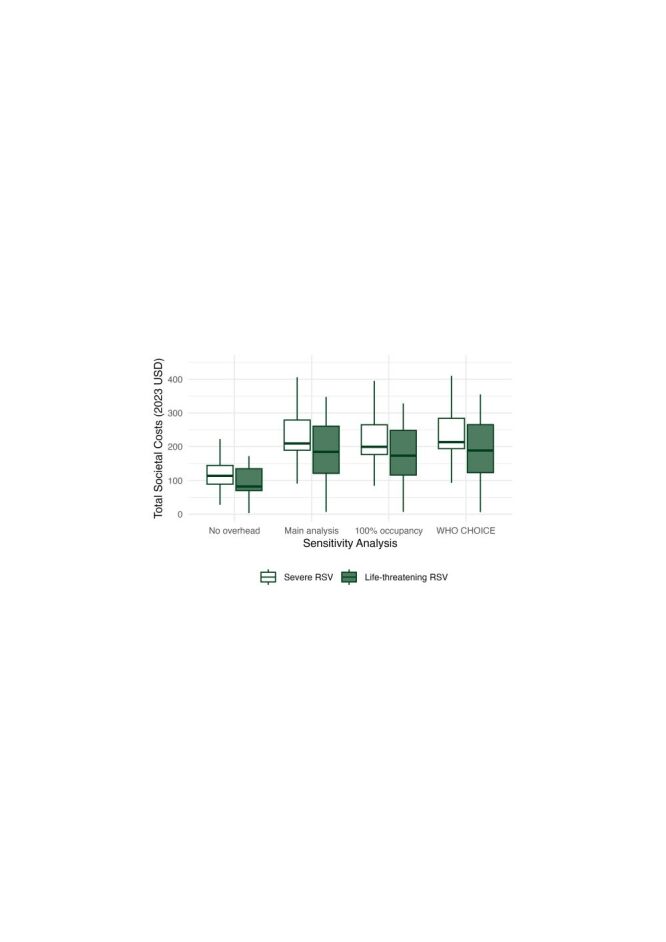
Total societal costs per episode of severe (admission, non-intensive care) and life-threatening (intensive care or fatality) RSV disease estimated in sensitivity analyses. Total societal costs included health system costs (direct medical; mainly overhead expenses) and household costs (out-of-pocket direct medical costs, direct non-medical costs, and indirect costs). From left to right: costs excluding overhead expenses (these costs are constant across all analyses); total costs including overhead expenses calculated for observed patient numbers assuming a median 7-day length of stay; total costs including overhead expenses calculated assuming 100% bed occupancy rate; total costs including WHO CHOICE estimated for health service delivery costs at tertiary facilities in Nigeria as a proxy for overhead expenses. Costs for non-severe (outpatient only) RSV are not shown due to small relative size (Table S3 in the **Online Supplementary Document**). Outlying observations are not plotted. RSV – respiratory syncytial virus, WHO CHOICE – World Health Organization CHOosing Interventions that are Cost-Effective.

Sensitivity analyses of the overhead expenses showed only slight variations in average costs of illness. Assuming 100% bed occupancy resulted in slightly lower overall costs (USD 232; 95% CI = 188–276 for severe RSV and USD 170; 95% CI = 115–226 for life-threatening RSV). When we used WHO CHOICE estimates for tertiary facilities as a proxy for overhead expenses, we estimated USD 12 (95% CI = 11–14) for non-severe RSV, USD 248 (95% CI = 201–295) for severe RSV, and USD 183 (95% CI = 122–243) for life-threatening RSV (**Figure 1**; Table S3 in the **Online Supplementary Document**).

## DISCUSSION

To our knowledge, this was the first prospective study to estimate the costs associated with one episode of RSV illness in Nigeria. We described the socioeconomic and clinical characteristics of children seeking care for RSV and non-RSV (S)ARI across three severity levels. From the societal, health system, and household perspectives, this study described the per-episode direct medical, direct non-medical, and indirect costs – therefore providing insight to the economic burden of RSV in Nigeria and contributing crucial data to our understanding of RSV health economics in underrepresented settings (LMICs). These data are needed to inform evaluations of the potential economic impact of introducing preventive interventions such as the long-acting mAb or maternal vaccine.

During this study, we captured one local RSV season in full and tested 792 children for RSV at a tertiary outpatient and inpatient facility in North-West Nigeria. We confirmed RSV infection in 19 and 32% of children <2 years old that sought care at the outpatient and inpatient facilities, respectively. Our study underlined the substantial burden of RSV in young children. In-hospital RSV positivity was consistent with other studies conducted previously at ABUTH [4,5].

RSV-related cost data are scarce in LMICs [22]. A Kenyan study describing inpatient costs of RSV in children under five years old was the only comparable study conducted in a fellow lower-middle-income sub-Saharan African country. Adjusted for inflation, mean RSV-associated costs in Kenya were USD 300 to USD 479 at two semi-rural referral hospitals between 2019 and 2021 [23]. These costs corresponded to 15% and 25% of the Kenyan gross domestic product (GDP) per capita in 2023, respectively [24]. In the current study, we estimated RSV inpatient care costs of USD 230 for children <2 years old, resulting in similar proportional results (14% of the 2023 Nigerian GDP per capita) as those reported for one of the Kenyan study hospitals [25]. Across the severity categories used in the current study, the costs averaged 1, 15, and 11% of the GDP per capita per episode of non-severe, severe, and life-threatening RSV.

We conducted sensitivity analyses to explore the effect of a potentially low bed occupancy rate, due to the rural setting of this study, on the estimated societal costs of illness. Assuming 100% bed occupancy yielded similar results as our main analyses with slightly lower per patient costs. Using WHO CHOICE estimates as a proxy for overhead expenses resulted in similar results as well. These findings support our estimated overhead expenses, and our results point to a substantial economic burden of RSV to the Nigerian health system.

Additionally, we found that childhood respiratory illness imposed substantial costs on households. Interviewed caregivers (mostly mothers) were often not employed. Few caregivers were accompanied by their household’s primary earner. In the absence of reliable household income data, the costs associated with an episode of RSV (S)ARI can be brought into context using the monthly minimum wage [26]. For non-severe RSV illness, the household costs per episode were 18% of the monthly Nigerian minimum wage (30 000 Nigerian naira (NGN); USD 50.73 [26]). This proportion was much higher for children with severe (241%) and life-threatening (173%) RSV. An overwhelming majority of caregivers (89%) reported using personal savings to cover the care costs, therefore signalling potentially catastrophic household costs for RSV care, further underscored by a low NHIS coverage in the study population.

Notably, the costs of care are influenced by access to care. With fewer available services come lower cost. Similarly, refusal of or withdrawal from care reduces the overall average cost of care. Care-seeking behaviour can affect costs directly (*e.g*. refusal or withdrawal) as well as through increased risk of severe health outcomes (*e.g*. due to late presentation). In this study, we observed some community mortality post discharge as well as among children who only received outpatient care. Costs were lower for children with life-threatening RSV than children with severe RSV. One explanation for this is the inclusion of children with fatal outcomes, who received little to no inpatient services in the life-threatened group. We also observed a significant difference in travel distance across severity groups, reflecting findings from a previous study that identified travel distance as a hurdle to timely care-seeking behaviour [27]. Comparisons in costs and median travel distance across the severe and life-threatened RSV cases, however, should be made carefully considering that this study was limited by small group sizes with increasing severity, which shrunk further in sensitivity analyses.

In this study, we did not collect sufficient data to describe caregiver withdrawal and refusal or socioeconomic, gender, and religious factors affecting timely care-seeking behaviour and access to care. Anecdotal information from local experts suggested, however, a financial hurdle to prolonged (intensive) care, leading to late intervention or withdrawal from care. This theory is supported by a previous cross-sectional study in North-West Nigeria that found respiratory symptoms (including breathing issues and chest indrawing) were not associated with care-seeking behaviour; whereas, socioeconomic factors such as education and wealth were [28]. These findings may provide a possible explanation for the mortality we observed outside the ICU.

This comprehensive study summarised the costs of RSV-(S)ARI in children <2 years old in North-West Nigeria, including sensitivity analyses that explored the impact of the study context on the results thus providing a crude estimate of RSV costs in the broader Nigerian context. Our findings reveal gaps in achieving universal health coverage in the study population, placing a large economic burden on households, and possibly affecting the timeliness and accessibility of care.

## CONCLUSIONS

In conclusion, we found costs of illness associated with one episode of RSV infection in children <2 years old to be substantial for both the health system and households in Nigeria. Future studies are needed to investigate challenges and opportunities within Nigeria’s health financing policy to achieve universal health coverage to alleviate the burden on households. Furthermore, this study contributes to our growing understanding of the economic burden of RSV in LMICs and can inform national and international policy makers in evaluating emerging pharmaceutical interventions for RSV prevention in Nigeria.

## Additional material


Online Supplementary Document
