# Supplementary material for: Estimating the economic burden of respiratory syncytial virus infection among children <2 years old seeking care in North-West Nigeria
Source: J Glob Health. 2025 Dec 12;15:04307. doi: 10.7189/jogh.15.04307 (PMC12699286; doi:10.7189/jogh.15.04307)
Supplement: Online Supplementary Document [file jogh-15-04307-s001.pdf]

Supplement to: Shaaban FL, Garba MA, Nguyen A, Pecenka C, Rave N, Bont LJ; RSV GOLD III – Health Economics Study Group. Estimating the economic burden of respiratory syncytial virus infection among children under two years of age seeking care in North-West Nigeria. J Glob Health. 2025;15:04307.

With the RSV GOLD III – Health Economics Study we evaluate costs associated with acute lower respiratory tract infections (LRTIs) in children <2 years old in four low- and middle-income countries (Ghana, Mozambique, Nepal, and Nigeria), during one local respiratory season. Here, we provide supplementary material related to the study conducted in Nigeria.

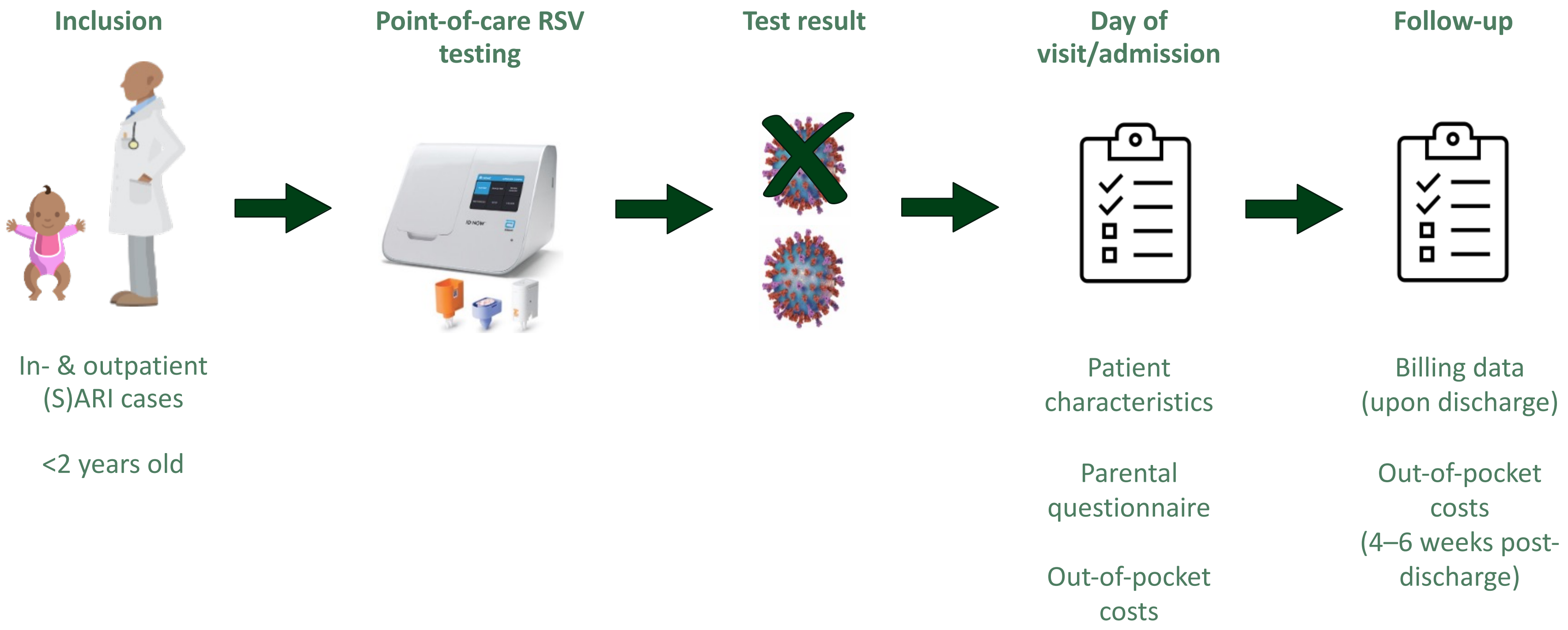

Figure S1. Study setup and data collection process. Follow-up was conducted over the telephone.
RSV – respiratory syncytial virus, (S)ARI – (severe) acute respiratory infection.

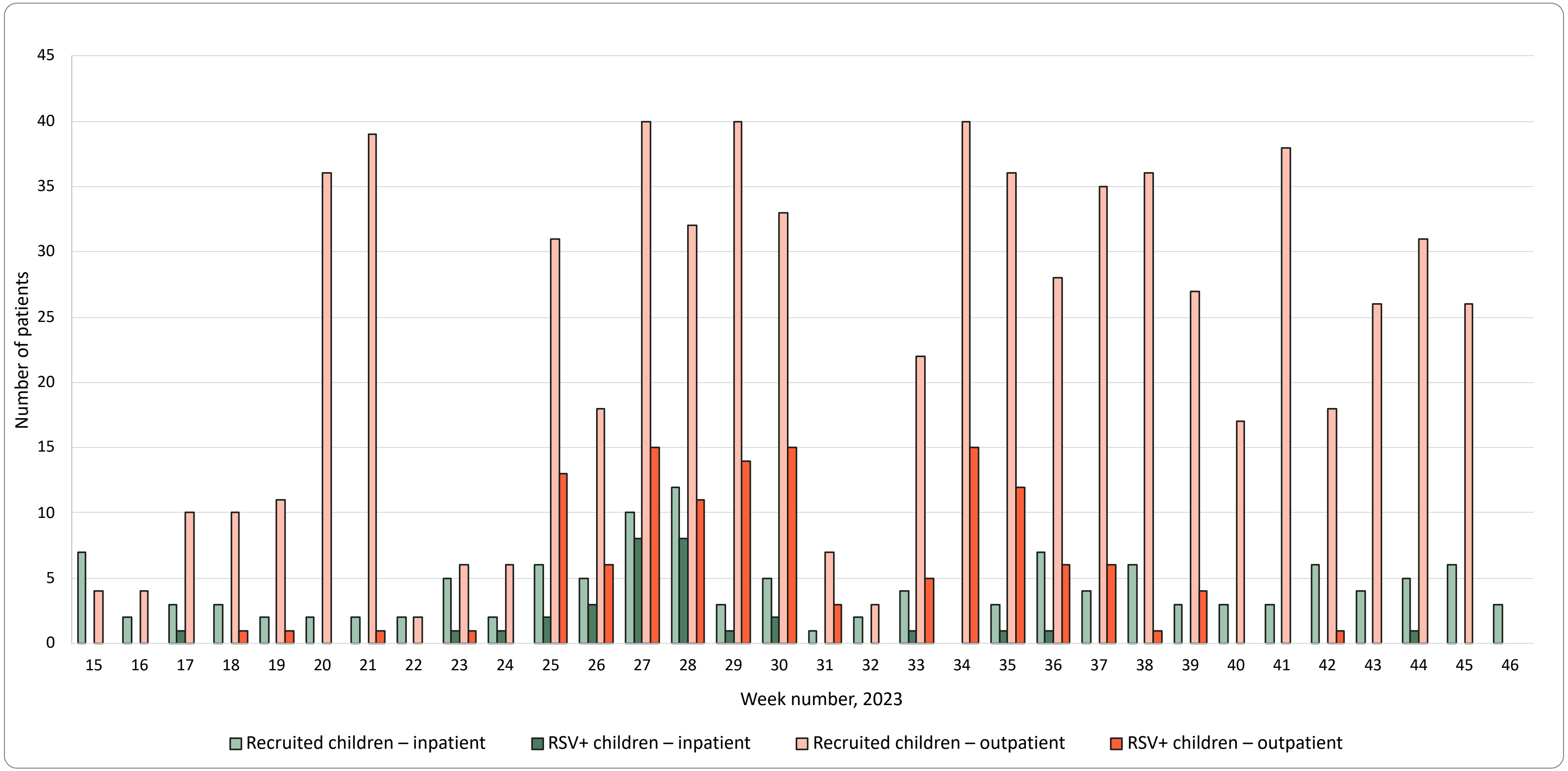

Figure S2. Weekly recruitment numbers during the study period at Ahmadu Bello University Teaching Hospital (inpatient) and Institute of Child Health Banzazzau (outpatient). The drop in observations during weeks 31–33 was the result of resident doctors' strikes. Due to depletion in testing materials, recruitment was closed two weeks prior to the planned end date. Low RSV activity after week 39, suggested a natural end of the season and no alternative testing arrangements were made.
RSV – respiratory syncytial virus.

Table S1. Overview of costs per LRTI episode among children <2 years old, by RSV and admission status, expressed in 2023 USD.

	RSV-positive (n=163)		RSV-negative (n=629)	
	**Outpatient (n=132)**	**Inpatient (n=31)**	**Outpatient (n=564)**	**Inpatient (n=65)**
**Societal costs total**				
x̅ (95% CI)	12.62 (11.19–14.04)	230.04 (192.96–267.13)	18.20 (16.24–20.15)	266.60 (222.67–310.53)
MD (IQR)	11.25 (9.35–13.37)	207.81 (178.49–289.22)	12.08 (9.96–16.43)	209.12 (180.74–274.16)
**Health system costs total***				
x̅ (95% CI)	3.65 (3.60–3.71)	115.89 (91.43–140.34)	3.63 (3.62–3.65)	129.78 (109.41–150.15)
MD (IQR)	3.62 (3.62–3.62)	110.32 (78.80–141.83)	3.62 (3.62–3.62)	94.56 (78.80–157.59)
**Household costs total**				
x̅ (95% CI)	8.96 (7.54–10.39)	114.16 (94.12–134.19)	14.56 (12.61–16.52)	136.82 (108.98–164.67)
MD (IQR)	7.63 (5.73–9.74)	107.44 (76.05–141.88)	8.45 (6.34–12.81)	104.95 (82.82–154.13)
Direct medical costs				
x̅ (95% CI)	6.77 (5.43–8.12)	88.95 (73.24–104.66)	10.08 (8.76–11.40)	95.87 (83.35–108.40)
MD (IQR)	5.37 (4.23–7.50)	80.58 (58.51–110.93)	6.09 (4.65–9.50)	83.89 (66.54–111.30)
Direct non-medical costs				
x̅ (95% CI)	1.69 (1.45–1.94)	15.83 (10.03–21.62)	3.13 (2.37–3.89)	30.12 (13.66–46.59)
MD (IQR)	1.35 (1.01–1.86)	8.03 (3.17–24.69)	1.35 (0.73–2.24)	14.20 (3.01–27.40)
Indirect costs				
x̅ (95% CI)	1.01 (0.81–1.22)	12.64 (8.68–16.60)	1.94 (1.46–2.41)	17.01 (10.89-23.13)
MD (IQR)	0.88 (0.59–1.18)	11.80 (0.59–18.88)	0.88 (0.59–1.47)	11.80 (5.03-18.95)

CI – confidence interval, IQR – interquartile range, LRTI – lower respiratory tract infection, MD – median, RSV – respiratory syncytial virus.
*Health system costs included direct medical costs only and consisted of hospitality/facility-based fees and costs covered by the national health insurance scheme. Due to the scheme's low coverage (0.8%), health systems costs approximated to the hospitality/facility-based fees. Therefore, health system costs are not specified by sub-category.

Table S2. Overview of costs per LRTI episode among children <2 years old, by RSV status and severity level, across selective sensitivity analyses, expressed in 2023 USD.

Full sample analysis (N=792):

	RSV-positive (n=163)			RSV-negative (n=629)		
	**Non-severe (n=130)**	**Severe (N=n=19)**	**Life-threatened (n=14)**	**Non-severe (n=562)**	**Severe (n=33)**	**Life-threatened (n=34)**
**Societal costs total**						
$\bar{x}$ *(95% CI)*	12.68 (11.20–14.16)	244.07 (197.77–290.36)	179.36 (120.23–238.50)	18.23 (16.31–20.15)	237.75 (205.32–270.18)	279.52 (201.23–357.81)
MD (IQR)	11.30 (9.37–13.39)	209.31 (188.34–289.22)	184.36 (117.17–278.21)	12.08 (9.96–16.44)	208.83 (196.56–271.93)	208.50 (166.69–327.82)
**Health system costs total**						
$\bar{x}$ *(95% CI)*	3.65 (3.59–3.71)	121.86 (89.53–154.19)	91.75 (56.77–126.72)	3.63 (3.62–3.65)	118.43 (100.13–136.74)	133.37 (98.24–168.50)
MD (IQR)	3.62 (3.62–3.62)	110.32 (78.80–126.08)	94.56 (47.28–142.51)	3.62 (3.62–3.62)	94.56 (78.80–141.83)	102.44 (78.80–173.35)
**Household costs total**						
$\bar{x}$ *(95% CI)*	9.03 (7.54–10.51)	122.21 (94.38–150.03)	87.62 (61.20–114.04)	14.59 (12.67–16.51)	119.32 (98.45–140.19)	146.15 (97.75–194.55)
MD (IQR)	7.67 (5.75–9.76)	114.17 (79.73–146.91)	82.34 (69.89–135.70)	8.45 (6.34–12.81	105.19 (87.92–130.03)	103.08 (72.34–154.47)

Reason for exclusion – length of stay was ≥10 days (N=768):

	RSV-positive (n=157)			RSV-negative (n=611)		
	**Non-severe (n=130)**	**Severe (n=16)**	**Life-threatened (n=11)**	**Non-severe (n=562)**	**Severe (n=25)**	**Life-threatened (n=24)**
**Societal costs total**						
$\bar{x}$ *(95% CI)*	N/A	217.48 (181.32–253.63)	135.44 (84.97–185.92)	N/A	206.47 (182.07–230.87)	166.77 (137.98–195.55)
MD (IQR)	N/A	206.01 (183.42–245.70)	154.72 (14.01–197.77)	N/A	204.39 (172.18–223.78)	181.03 (154.91–210.39)
**Health system costs total**						
$\bar{x}$ *(95% CI)*	N/A	98.39 (87.59–109.19)	65.19 (37.51–92.87)	N/A	91.40 (82.11–100.70)	79.73 (62.81–96.66)
MD (IQR)	N/A	102.44 (78.80–110.32)	63.04 (3.62–110.32)	N/A	94.56 (78.80–110.32)	86.68 (63.04–117.93)
**Household costs total**						
$\bar{x}$ *(95% CI)*	N/A	119.09 (87.37–150.81)	70.25 (44.59–95.91)	N/A	115.07 (94.83–135.31)	87.03 (70.36–103.71)
MD (IQR)	N/A	103.48 (77.89–142.62)	71.36 (14.01–107.44)	N/A	106.92 (92.02–126.19)	89.42 (70.01–105.09)

Table S2. Continued

Reason for exclusion – missing billing data (N=777):

	RSV-positive (n=161)			RSV-negative (n=616)		
	**Non-severe (n=130)**	**Severe (n=19)**	**Life-threatened (n=12)**	**Non-severe (n=553)**	**Severe (n=32)**	**Life-threatened (n=31)**
**Societal costs total**						
$\bar{x}$ (95% CI)	12.68 (11.20–14.16)	244.07 (197.77–290.36)	207.52 (153.50– 261.54)	17.65 (15.81–19.48)	241.83 (209.36–274.29)	288.13 (206.61–369.65)
MD (IQR)	11.30 (9.37–13.39)	209.31 (188.34–289.22)	192.66 (144.56–303.08)	12.08 (9.96–16.44)	204.39 (172.18–223.78)	181.03 (154.91–210.39)
**Health system costs total**						
$\bar{x}$ (95% CI)	3.65 (3.59–3.71)	121.86 (89.53–154.19)	106.73 (73.82–139.65)	3.63 (3.62–3.65)	119.18 (99.51–138.85)	135.99 (100.28–171.70)
MD (IQR)	3.62 (3.63–3.62)	110.32 (78.80–126.08)	110.32 (55.16–157.93)	3.62 (3.63–3.62)	94.56 (78.80–149.71)	110.32 (78.80–173.35)
**Household costs total**						
$\bar{x}$ (95% CI)	9.03 (7.54–10.51)	122.21 (94.38–150.03)	100.79 (76.62–124.95)	14.01 (12.18–15.84)	122.65 (102.78–142.52)	152.13 (100.16–204.10)
MD (IQR)	7.67 (5.75–9.76)	114.17 (79.73–146.91)	97.45 (71.11–137.27)	8.43 (6.34–12.75)	106.05 (89.00–141.74)	103.65 (77.38–154.47)

Reason for exclusion – missing billing data or was outpatient fatality (N=775):

	RSV-positive (n=160)			RSV-negative (n=615)		
	**Non-severe (n=130)**	**Severe (n=19)**	**Life-threatened (n=11)**	**Non-severe (n=553)**	**Severe (n=32)**	**Life-threatened (n=30)**
**Societal costs total**						
$\bar{x}$ (95% CI)	N/A	N/A	225.46 (176.87–274.05)	N/A	N/A	297.25 (215.89–378.61)
MD (IQR)	N/A	N/A	197.77 (154.72–327.96)	N/A	N/A	210.39 (169.98–327.82)
**Health system costs total**						
$\bar{x}$ (95% CI)	N/A	N/A	116.11 (84.28–147.94)	N/A	N/A	140.41 (105.44–175.37)
MD (IQR)	N/A	N/A	110.32 (63.04–173.35)	N/A	N/A	117.93 (78.80–173.35)
**Household costs total**						
$\bar{x}$ (95% CI)	N/A	N/A	109.35 (89.02–129.68)	N/A	N/A	156.84 (103.96–209.71)
MD (IQR)	N/A	N/A	107.44 (71.36–138.84)	N/A	N/A	104.30 (81.80–154.47)

CI – confidence interval, IQR – interquartile range, LRTI – lower respiratory tract infection, MD – median, RSV – respiratory syncytial virus.

Table S3. Overview of total societal costs per LRTI episode among children <2 years old, by RSV status and severity level, across sensitivity analyses of overhead expenses, expressed in 2023 USD.

	RSV positive (n=163)			RSV negative (n=629)		
	Non-severe (n=130)	Severe (n=19)	Life-threatened (n=14)	Non-severe (n=562)	Severe (n=33)	Life-threatened (n=34)
**No overhead expenses**						
$\bar{x}$ (95% CI)	9.06 (7.58–10.53)	126.28 (97.51–155.06)	87.67 (61.23–114.11)	14.60 (12.68–16.52)	119.32 (98.45–140.19)	146.74 (98.39–195.10)
MD (IQR)	7.67 (5.75–9.76)	114.17 (79.73–146.91)	82.34 (69.89–136.38)	8.45 (6.34–12.81)	105.19 (87.92–130.03)	103.08 (72.34–156.43)
**Observed patient numbers**						
$\bar{x}$ (95% CI)	12.68 (11.20–14.16)	244.07 (197.77–290.36)	179.36 (120.23–238.50)	18.23 (16.31–20.15)	237.75 (205.32–270.18)	279.52 (201.23–357.81)
MD (IQR)	11.30 (9.37–13.39)	209.31 (188.34–289.22)	184.36 (117.17–278.21)	12.08 (9.96–16.44)	208.83 (196.56–271.93)	208.50 (166.69–327.82)
**100% bed occupancy**						
$\bar{x}$ (95% CI)	N/A	232.36 (188.23–276.49)	170.30 (114.58–226.02)	N/A	225.98 (194.99–256.97)	266.35 (191.24–341.46)
MD (IQR)	N/A	199.32 (174.24–271.99)	173.39 (112.47–264.12)	N/A	201.00 (188.73–253.13)	197.24 (160.42–310.59)
**WHO CHOICE estimates**						
$\bar{x}$ (95% CI)	12.32 (10.85–13.80)	248.34 (201.24–295.44)	182.62 (122.20–243.04)	17.87 (15.95–19.79)	242.05 (209.09–275.02)	284.31 (204.84–363.78)
MD (IQR)	10.94 (9.02–13.03)	213.32 (193.49–295.51)	188.36 (118.89–283.36)	11.72 (9.61–16.08)	211.69 (201.50–276.45)	212.50 (168.97–334.12)

CI – confidence interval, IQR – interquartile range, LRTI – lower respiratory tract infection, MD – median, RSV – respiratory syncytial virus, WHO CHOICE – World Health Organization CHOosing Interventions that are Cost-Effective.

RSV GOLD III – Health Economics Study Group members*

Cameroon:

Frédéric Debellut – Center for Vaccine Innovation and Access, PATH, Geneva, Switzerland

Norbert Fuhngwa – Triangle Research Foundation, Douala, Cameroon

Henshaw Mandi – Triangle Research Foundation, Douala, Cameroon

Ghana:

Rosemary Akuaku – Department of Child Health, Korle Bu Teaching Hospital, Accra, Ghana

Joycelyn Dame – Department of Child Health, University of Ghana Medical School Korle Bu Teaching Hospital, Accra, Ghana

Amma Ekem – Department of Child Health, Korle Bu Teaching Hospital, Accra, Ghana

Bamenla Goka – Department of Child Health, University of Ghana Medical School Korle Bu Teaching Hospital, Accra, Ghana

Ebenezer Ntow – Department of Child Health, Korle Bu Teaching Hospital, Accra, Ghana

Kwabena A. Osman – Department of Child Health, University of Ghana Medical School Korle Bu Teaching Hospital, Accra, Ghana

Mozambique:

Assucênio Chissaque – Instituto Nacional de Saúde, Marracuene district, Maputo, Mozambique; Instituto de Higiene e Medicina Tropical, Universidade Nova de Lisboa, Lisbon, Portugal

Nilsa de Deus – Instituto Nacional de Saúde, Marracuene district, Maputo, Mozambique

Esperança Lourenço Guimarães – Instituto Nacional de Saúde, Marracuene district, Maputo, Mozambique; Instituto de Higiene e Medicina Tropical, Universidade Nova de Lisboa, Lisbon, Portugal

Braiton Maculuve – Ministério da Saúde, Maputo, Mozambique

Elias Manjate – Faculty of Medicine, University Eduardo Mondlane, Maputo, Mozambique

Yara Manjate – Faculty of Medicine, University Eduardo Mondlane, Maputo, Mozambique

Izilda Matimbe – Faculty of Medicine, University Eduardo Mondlane, Maputo, Mozambique

Tufária Mussá – Faculty of Medicine, University Eduardo Mondlane, Maputo, Mozambique

Mirela Pale – Instituto Nacional de Saúde, Marracuene district, Maputo, Mozambique

Cesar Palha – Faculty of Medicine, University Eduardo Mondlane, Maputo, Mozambique

Cristina Sinussene – Faculty of Medicine, University Eduardo Mondlane, Maputo, Mozambique
Farida Zavala – Faculty of Medicine, University Eduardo Mondlane, Maputo, Mozambique

Nepal:
Ram H. Chapagain – Kanti Children's Hospital, Kathmandu, Nepal; Nepal Paediatrics Society, Kathmandu, Nepal
Rita Dhital – Nepal Paediatrics Society, Kathmandu, Nepal
Upendra Dhungana – Public Health Administrator; Ministry of Health and Population. Nepal
Prakash Joshi – Kanti Children's Hospital, Kathmandu, Nepal; Nepal Paediatrics Society, Kathmandu, Nepal
Ranju Karki – Nepal Paediatrics Society, Kathmandu, Nepal
Adita Nepali – Nepal Paediatrics Society, Kathmandu, Nepal
Uttam Paudel – Post Doctorate Researcher (Health Economics), Chulalongkorn University
Arun K. Sharma – Tribhuvan University Teaching Hospital, Institute of Medicine, Kathmandu, Nepal; Nepal Paediatrics Society, Kathmandu, Nepal
Rupesh Shrestha – Tribhuvan University Teaching Hospital, Institute of Medicine, Kathmandu, Nepal
Nirasta Thakili – Nepal Paediatrics Society, Kathmandu, Nepal

Nigeria:
Fadlulai Abdu-Raheem – Department of Paediatrics, Ahmadu Bello University Teaching Hospital, Zaria, Nigeria
Anas Abubakar – Department of Paediatrics, Ahmadu Bello University Teaching Hospital, Zaria, Nigeria
Abdullahi Aminu – Department of Paediatrics, Ahmadu Bello University Teaching Hospital, Zaria, Nigeria
Maria A. Garba – Department of Paediatrics, Ahmadu Bello University Teaching Hospital, Zaria, Nigeria
Fatima J. Giwa – Department of Medical Microbiology, Ahmadu Bello University Teaching Hospital, Zaria, Nigeria
Habiba Lawal – Institute of Child Health, Ahmadu Bello University, Banzazzau, Zaria, Nigeria
Bernsah D. Lawong – Department of Economics, Ahmadu Bello University, Zaria, Nigeria
Abdullahi Musa – Department of Paediatrics, Ahmadu Bello University Teaching Hospital, Zaria, Nigeria
Teddy Naddumba – Center for Vaccine Innovation and Access, PATH, Kampala, Uganda
Aira A. Olorukooba – Department of Paediatrics, Ahmadu Bello University Teaching Hospital, Zaria, Nigeria

Support:
Andrew Clark – Department of Health Services Research and Policy, London School of Hygiene & Tropical Medicine, London, UK
An Nguyen – Center for Vaccine Innovation and Access, PATH, Ho Chi Minh city, Vietnam
Clint Pecenka – Center for Vaccine Innovation and Access, PATH, Seattle, WA, USA

The Netherlands:
Louis J. Bont – University Medical Centre Utrecht, Utrecht, The Netherlands
Neele Rave – University Medical Centre Utrecht, Utrecht, The Netherlands
Farina L. Shaaban – University Medical Centre Utrecht, Utrecht, The Netherlands

*The authors are listed in alphabetical order of their surnames, according to the specific country teams through which they were involved in the study. Team members from University Medical Centre Utrecht, along with supporting staff, were involved at all study sites. A detailed overview of authorship contributions for each country can be found in each respective article.
